# Chronic obstructive pulmonary disease (COPD) and occupational exposures

**DOI:** 10.1186/1745-6673-1-11

**Published:** 2006-06-07

**Authors:** Piera Boschetto, Sonia Quintavalle, Deborah Miotto, Natalina Lo Cascio, Elena Zeni, Cristina E Mapp

**Affiliations:** 1 Department of Experimental and Clinical Medicine, University of Ferrara, Ferrara, Italy

## Abstract

Chronic obstructive pulmonary disease (COPD) is one of the leading causes of morbidity and mortality in both industrialized and developing countries.

Cigarette smoking is the major risk factor for COPD. However, relevant information from the literature published within the last years, either on general population samples or on workplaces, indicate that about 15% of all cases of COPD is work-related.

Specific settings and agents are quoted which have been indicated or confirmed as linked to COPD. Coal miners, hard-rock miners, tunnel workers, concrete-manufacturing workers, nonmining industrial workers have been shown to be at highest risk for developing COPD.

Further evidence that occupational agents are capable of inducing COPD comes from experimental studies, particularly in animal models.

In conclusion, occupational exposure to dusts, chemicals, gases should be considered an established, or supported by good evidence, risk factor for developing COPD. The implications of this substantial occupational contribution to COPD must be considered in research planning, in public policy decision-making, and in clinical practice.

## 

1. Definition

2. Occupational exposures and COPD: epidemiologic evidence

3. Occupational exposures and COPD: experimental evidence

4. Occupationally-related COPD: diagnosis

5. Occupationally-related COPD: management and prevention

## 1. Definition

Chronic obstructive pulmonary disease (COPD) is a disease state characterized by airflow limitation that is not fully reversible. The airflow limitation is usually both progressive and associated with an abnormal inflammatory response of the lungs to noxious particles and gases [1].

Many previous definitions of COPD have emphasized the terms "emphysema" and "chronic bronchitis" which are no longer included in the definition of COPD [1]. Emphysema, or destruction of the gas-exchanging surface of the lung (alveoli), is a pathological term that is often (but incorrectly) used clinically and describes only one of several structural abnormalities present in patients with COPD. Chronic bronchitis, or the presence of cough and sputum production for at least 3 months in each of two consecutive years, remains a clinically and epidemiologically useful term. However, it does not reflect the major impact of airflow limitation on morbidity and mortality in COPD patients. It is also important to recognize that cough and sputum production may precede the development of airflow limitation; conversely, some patients develop airflow limitation without chronic cough and sputum production.

COPD does not have a clinical subcategory that is clearly identified as occupational, largely because the condition develops slowly and, given that the airflow limitation is chronic, does not reverse when exposure is discontinued. Thus, a clinical diagnosis of occupational COPD, using methods similar to those employed for occupational asthma, is not feasible. Epidemiologically, the identification of occupational COPD is based on observing excess occurrence of COPD among exposed workers [2-4].

Some work-related obstructive airway disorders have been classified as COPD but do not neatly fit into this category. For example, work-related variable airflow limitation may occur with occupational exposure to organic dusts such as cotton, flax, hemp, jute, sisal, and various grains. Such organic dust-induced airway disease is sometimes classified as an asthma-like disorder [5], but both chronic bronchitis and poorly reversible airflow limitation can develop with chronic exposure. Bronchiolitis obliterans and irritant-induced asthma are two other conditions that may overlap clinically with work-related COPD.

## 2. Occupational exposures and COPD: epidemiologic evidence

COPD is a major cause of chronic morbidity and mortality throughout the world. Many people suffer from this disease for years and die prematurely from it or its complications. COPD is currently the fourth leading cause of death in the world [6], and further increases in its prevalence and mortality can be predicted in the coming decades [7].

Cigarette smoking is undoubtedly the main cause of COPD in the population. A dose-response relationship between the amount smoked and an observed accelerated decline in ventilatory function have been consistently found in longitudinal epidemiological studies [1,8-11]; however, there is a huge individual variation. Fletcher and Peto [12], in an 8-yr prospective study of working men in West London, showed that the average decline in FEV_1 _in smokers is faster (60 ml/yr) than in non-smokers (30 ml/yr). However, smokers who develop COPD have an average decline in FEV_1 _of greater than 60 ml/yr, and only 15 to 20% of smokers develop clinically significant COPD. In addition, an estimated 6% of persons who had COPD in the United States are never smokers [13]. Cigarette smoke is analogous to a mixed inhalation exposure at a workplace because it is a complex mixture of particles and gases.

Despite the difficulty of disentangling the effect of cigarette smoke from those of other exposures, there is growing evidence from large population based studies suggesting that a sizeable proportion of the cases of COPD in a society may be attributable to workplace exposures to dusts, noxious gases/vapours, and fumes (DGVFs). The fraction of cases in a population that arise because of certain exposures is called the attributable fraction in the population or the population attributable risk (PAR). The American Thoracic Society (ATS) recently produced a consensus statement based on an evaluation of a number of large scale general population studies, and calculated that PAR for COPD was about 15% [14]. Several recent papers published since the completion of the ATS statement provide further evidence in support of a major contribution of occupational exposure to the burden of COPD. Hnizdo and coworkers from the National Institute for Occupational Safety and Health used data collected in the US population-based Third National Health and Nutrition Examination Survey on more than 9800 subjects to estimate the PAR for COPD attributable to work [15]. The analysis was adjusted for multiple factors, including smoking history. The industries with increased risk include rubber, plastics, and leather manufacturing, utilities, building services, textile manufacturing, and construction. The PAR for COPD attributable to work was estimated at 19% overall and 31% among never smokers. A second US population-based study conducted by Trupin and coworkers [16] obtained survey information on more than 2000 subjects. Occupational exposures were associated with increased risk of COPD after adjustment for smoking history and demographic variables. The PAR for COPD caused by these exposures was 20%. In this study, the PAR for combined current and former smokers was 56%. Smoking and occupational exposures to dusts, gases, and/or fumes had greater than additive effects. A third study from Sweden was designed to determine whether occupational exposure to dust, fumes, or gases, especially among never-smokers, increased the mortality from COPD [17]. A cohort of more than 317000 Swedish male construction workers was followed from 1971 to 1999. Exposure to inorganic dusts, gases and irritant chemicals, fumes, and wood dusts was based on a job-exposure matrix. An internal control group with "unexposed" construction workers was used, and the analyses were adjusted for age and smoking. There was a statistically significant increase mortality from COPD among those with any airborne exposure (relative risk 1.12). In a Poisson regression model, including smoking, age and the four major exposure groups listed previously, exposure to inorganic dust was associated with an increased risk, especially among never-smokers. The fraction of COPD among the exposed attributable to any airborne exposure was estimated as 10.7% overall and 52.6% among never-smokers. Thus, occupational exposure among construction workers increases mortality due to chronic obstructive pulmonary disease, even among never-smokers.

The determination of the PAR% due to occupational exposure has been complicated until recently by the lack of standardization of definition for COPD. Moreover, relatively few studies have been conducted with the specific purpose of determining the occupational contribution to COPD in the general population. In the studies that have been performed, there has been no consistency in terms of a strict definition of COPD. Some have presented data on symptoms and diseases, others have presented data on lung function, and a few have done both. Although a certain degree of standardization has been accomplished for cough and phlegm, dyspnea has been defined more variably among the studies.

While cigarette smoking and occupational exposures appear to account in combination for the major proportion of the population attributable risk of COPD, other influences are potentially important. The understanding of genetic susceptibility to this condition is still in its relative infancy, but certain data do suggest that genetics influences may be important [18], when considering both the established disease and the accelerated annual decline in FEV_1_. Furthermore, interactions have been noted between α_1 _anti-trypsin deficiency and environmental exposures in the development of COPD [19].

## 3. Occupational exposures and COPD: experimental evidence

The airflow limitation that defines COPD is associated with lesions that obstruct the small conducting airways, produce emphysematous destruction of the lung's elastic recoil force with closure of small airways, or both [20]. Experimental studies have demonstrated that several agents, including sulphur dioxide, mineral dusts, vanadium and endotoxin, are capable of inducing chronic obstructive bronchitis in animal models [21-24]. The list of agents that can cause emphysema in animals includes several for which there is also epidemiological evidence in exposed occupational cohorts, such as cadmium, coal, endotoxin, and silica [25]. The clearest human model of emphysema is that of α_1 _anti-trypsin deficiency [protease inhibitor phenotype Z (PI*Z)] [26]. This phenotype affects only a small percentage of the general population and is responsible for a correspondingly small fraction of the total burden of COPD. Although smoking is the most potent and well-established cofactor in emphysema related to α_1 _anti-trypsin deficiency, occupational exposure are linked to such disease as well [27,28].

Because α_1 _anti-trypsin is the endogenous inhibitor of neutrophil elastase and neutrophil elastase is capable to cause alveolar destruction, it has long been considered the major player in the development of emphysema. Yet, despite these evidences, it has been difficult to convincingly establish a role for neutrophil elastase in emphysema. The association of neutrophil elastase with human emphysema has been inconsistent, the extracellular release of neutrophil elastase has been questioned, and other proteinases have been shown to play a role in experimental models of emphysema. The finding that a murine knockout model lacking macrophage metalloelastase (MME) is resistant to the development of cigarette smoke-induced emphysema has created great interest in this enzyme and in the potential importance of other proteases [29-31].

The occupationally relevant agents that can cause emphysema (cadmium, coal, endotoxin, and silica), all cause the centrilobular form of the disease rather than the panacinar form that is associated with α_1 _anti-trypsin deficiency so mechanisms other than uninhibited neutrophil elastase activity are likely operative. The recent evidence about MME suggests a potential mechanism by which inhaled dusts or fumes could cause emphysema since macrophages have a primary role in the clearance of these materials from the terminal airways and alveoli.

## 4. Occupationally-related COPD: diagnosis

Cigarette smoking is by far the predominant risk factor for COPD. Till today, diagnostic assessments able to calculate the relative contribution of work exposures in a smoker with COPD are not available. However, adjustment of associations between occupational exposure and COPD for smoking status has been performed in epidemiological studies, showing that occupational risks likely play a role on their own. Thus, physicians must be aware of the potential occupational aetiologies for obstructive airway disease and should consider them in every patient with COPD. An occupational history should be the first step in the initial evaluation of the patient. A proper occupational history consists of a chronological list of all jobs, including job title, a description of the job activities, potential toxins at each job, and an assessment of the extent and duration of exposure. The length of time exposed to the agent, the use of personal protective equipment such as respirators, and a description of the ventilation and overall hygiene of the workplace are helpful in attempting to quantify exposure from the patient's history.

Additional information can be obtained from a visit to the workplace by experts in occupational hygiene, from material safety data sheets for workplace chemicals, and from the manufacturers of the workplace substances.

Identifying occupational risk factors on the individual level is important for prevention of disease before it is advanced and for modifying disability risk once disease is established [32]. In addition, the clinician has a critical role in case identification for the purposes of public health surveillance and appropriate work-related insurance compensation.

## 6. Occupationally-related COPD: management and prevention

Directions about the management and prevention of work-related diseases [33-35], can be applied to COPD as well. Physicians should attempt to understand the patient's occupational exposure and whether he/she has been adequately trained in the dangers of these exposures and how to manage them. Removal of the respiratory irritants and substitution of non-toxic agents are the best approach because they eliminate the work-related COPD hazard. If substitution is not possible, ongoing maintenance of engineering controls, such as enclosure of the industrial process and improving work area ventilation, are useful. Administrative controls (e.g., transfer to another job or change in work practices), and personal protective equipment (e.g., masks or respirators) should be mentioned, although less effective in decreasing exposures to respiratory tract irritants.

Guidelines for identification and management of individuals with work-related asthma have been recently published [36] and are relevant to work-related COPD. Unlike workers with sensitizer-induced asthma, workers with irritant-induced asthma or COPD may continue to work in their usual jobs if their exposure to the inciting agent is diminished via proper engineering controls or respiratory protective equipment if engineering controls are not feasible.

Prevention must be the primary tool for decreasing the incidence of morbidity and disability from work-related COPD, which can become severely disabling disease.

Primary prevention is designed to abate hazards before any damage or injury has occurred. Primary prevention strategies encompass the same exposure controls (elimination, engineering controls, administrative controls, personal protective equipment) described for management of work-related asthma and COPD due to irritant exposure. As cigarette smoking is the main risk factor for COPD, we wish to stress that smoking should be discouraged outside the workplace as well as inside the workplace.

Secondary prevention addresses early detection of the disease so that its duration and severity can be minimized. Medical surveillance programs are a type of secondary prevention. For medical surveillance of COPD, short symptom questionnaires can be administered before employment and repeated annually. They should include items such as improvement in respiratory symptoms on week-ends and holidays [37-39]. In addition, spirometry can be performed on an annual basis and compared to baseline spirometric testing at the time of hire. Review of peak expiratory flow rate records over several weeks can also detect workers at risk for developing irritant-induced COPD.

Tertiary prevention aims at the prevention of permanent COPD. It includes institution of appropriate health care. Furthermore, early recognition of the disease and early removal from, or reduction of, exposure, make it more likely that the patient will avoid permanent COPD.

Public policy needs to be better informed about the roles of occupational factors in obstructive airway disease. This will require active education and outreach on the part of the medical-scientific community. Specific public policy issues to be re-examined in light of the magnitude of the occupational contribution to the burden of airway disease include standard setting for exposure in and out of the workplace, attribution criteria for compensation, health care costs and their assignment, and health care resources allocation.

The clinician must be aware of the potential occupational aetiologies for obstructive airway disease and consider them in every patients with asthma or COPD. Identifying occupational risk factors on the individual level is important for prevention of disease before it is advanced and for modifying disability risk once disease is established [32]. In addition, the clinician has a critical role in case identification for the purposes of public health surveillance and appropriate work-related insurance compensation.

## Conclusion

Careful review of the literature demonstrated that approximately 15% of COPD is work-related and that new agents causing COPD, as well as new cases with persistent airflow limitation associated with work, are still being reported. It definitely supports the concept that in a new classification of risk factors for COPD, occupational exposure to dusts, chemicals, gases should be considered an established, or supported by good evidence, risk factor.

Besides epidemiological studies, further experimental studies can lead to a better understanding of the occupational hazards which may cause COPD and establish a stronger link between the severity of COPD and specific occupations. Experimental studies may actually serve as models from which to derive basic insights of COPD and to identify a cellular basis of the work-related disease.

## Authors' contributions

PB, SQ, DM, NLC, EZ and CEM have all been involved in drafting the article or revising it critically for important intellectual content and have given final approval of the version to be published.

## Declaration of competing interests

The author(s) declare that they have no competing interests.
